# An End-to-End Computationally Lightweight Vision-Based Grasping System for Grocery Items

**DOI:** 10.3390/s25175309

**Published:** 2025-08-26

**Authors:** Thanavin Mansakul, Gilbert Tang, Phil Webb, Jamie Rice, Daniel Oakley, James Fowler

**Affiliations:** Centre for Robotics and Assembly, Faculty of Engineering and Applied Sciences, Cranfield University, Bedford MK43 0AL, UK; g.tang@cranfield.ac.uk (G.T.); p.f.webb@cranfield.ac.uk (P.W.); jamie.rice@cranfield.ac.uk (J.R.); daniel.oakley@cranfield.ac.uk (D.O.); j.fowler@cranfield.ac.uk (J.F.)

**Keywords:** vision-based grasping system, end-to-end grasp detection, mobile manipulator, lightweight computation, object detection, object pose estimation, machine vision

## Abstract

Vision-based grasping for mobile manipulators poses significant challenges in machine perception, computational efficiency, and real-world deployment. This study presents a computationally lightweight, end-to-end grasp detection framework that integrates object detection, object pose estimation, and grasp point prediction for a mobile manipulator equipped with a parallel gripper. A transformation model is developed to map coordinates from the image frame to the robot frame, enabling accurate manipulation. To evaluate system performance, a benchmark and a dataset tailored to pick-and-pack grocery tasks are introduced. Experimental validation demonstrates an average execution time of under 5 s on an edge device, achieving a 100% success rate on Level 1 and 96% on Level 2 of the benchmark. Additionally, the system achieves an average compute-to-speed ratio of 0.0130, highlighting its energy efficiency. The proposed framework offers a practical, robust, and efficient solution for lightweight robotic applications in real-world environments.

## 1. Introduction

The global demand for online grocery ordering and delivery has seen rapid growth, particularly following the COVID-19 pandemic. As consumers increasingly seek convenience and time efficiency, the online grocery market is projected to grow at a compound annual growth rate (CAGR) of 25.3% from 2022 to 2030, reaching an estimated market size of approximately USD 1800 billion [1]. This shift reflects a broader transformation in consumer behavior, where digital solutions offer advantages over traditional in-store shopping, including reduced travel time, lower transportation costs, and simplified purchasing experiences.

Despite the advantages of door-to-door delivery, common issues such as incorrect item load and delivery delays persist. These challenges are often the result of human error and inefficiencies in operational procedures. For the retail industry, maintaining operational efficiency and ensuring high customer satisfaction are critical to business success. At the same time, the sector is increasingly confronted with labor shortages and rising operational costs, challenges that are expected to intensify in the near future [2].

To address these issues, robotic automation offers a promising solution. Rather than replacing human workers, robots can support and collaborate with them, enhancing productivity through human–robot interaction. Grocery delivery operations involve a range of repetitive and time-sensitive tasks, where robotic assistance can significantly reduce order errors, improve delivery predictability, lower operational costs, and streamline workflows [3].

From a research perspective, identifying the most suitable robotic system for this application presents a key challenge. Given the task requirements, such as navigating to the correct shelf, identifying and locating ordered items, picking them accurately, and placing them in a designated delivery area, a mobile manipulator emerges as an ideal solution. With its combined mobility and manipulation capabilities, the mobile manipulator can effectively fulfill the sophisticated demands of automated grocery delivery.

The development and deployment of mobile manipulators have played a crucial role in robotics. These systems provide both flexibility and improved task efficiency. However, achieving a truly smart industry requires advancements in several areas, including perception, adaptability to unstructured environments, and computational efficiency [4].

In robot sensing, machine vision is a fundamental component because it provides rich information similar to human vision. However, it still falls short of human-like capabilities. Ideally, a robot should be able to perceive its surroundings, process input data, make decisions, and perform tasks effectively in unstructured environments. Shahria et al. [5] highlight the importance of vision-based robotic approaches and emphasize the need to improve performance, stability, robustness, and processing speed to achieve real-time autonomous systems.

Intriguingly, there is a trade-off between performance and speed. High-performance systems prioritize accuracy by using complex processes and networks, which require significant computational power, expensive hardware, and high energy consumption. In contrast, high-speed systems focus on reducing execution time by using simpler and more efficient structures, such as traditional methods. To balance both performance and speed, it is essential to evaluate different methodologies and optimize key components. For example, a vision-based grasping system consists of three main parts: object detection, object pose estimation, and grasp estimation. Each of these elements can be assessed, improved, and refined to enhance real-world applicability and achieve real-time performance.

### 1.1. Related Work

#### 1.1.1. Grasp Detection

A comprehensive review of vision-based robotic grasping systems [6] classifies the grasp detection task into three fundamental components: (1) object localization, (2) object pose estimation, and (3) grasp estimation. Numerous approaches have been proposed for each component, ranging from traditional algorithms to recent state-of-the-art techniques. These developments provide a broad foundation and advanced insights into robotic perception; however, no single method has yet demonstrated universal applicability across all use cases. For instance, a mobile manipulator employing 3D perception, edge detection, and a genetic optimization algorithm performs well for depalletizing box-shaped objects under laboratory conditions [7], but it remains to be validated in dynamic industrial settings or with more complex objects such as bottles and cans. These limitations emphasize that grasping solutions must be tailored based on the specific object types, operating environments, performance requirements, and system constraints.

In pick-and-pack grocery applications, most objects exhibit relatively simple geometric shapes such as boxes, cylinders, and spheres. However, certain items, such as spray bottles, scissors, power tools, and rolls of tape, introduce greater geometric complexity and present additional challenges for robotic grasping. The operational environment is typically a supermarket, where products are arranged on shelves and aisles may be partially obstructed by customers or other dynamic elements. To function effectively in such real-world scenarios, mobile manipulators must be compact, computationally efficient, and energy-aware. Achieving these capabilities necessitates a thorough examination of existing limitations and the exploration of emerging opportunities in system architecture, perception, and motion planning.

The key challenges and research gaps in this domain are outlined in [8], particularly the trade-off between accuracy and processing speed. While recent works attempt to maximize task success or detection precision, they often overlook computational constraints. Maintaining high accuracy while improving speed requires trimming redundant operations and retaining only essential modules. This is especially relevant for robotic systems operating under resource limitations, such as mobile manipulators deployed in real-world environments.

Moreover, the importance of computationally lightweight systems is increasing. Vision-based robotic systems, especially those that incorporate deep learning, often demand powerful hardware due to the complexity of convolutional neural networks and the need for large and high-quality datasets. These requirements lead to high computational loads and significant memory usage. However, by shifting the focus toward efficient computation, researchers can reduce the dependence on high-end CPUs, GPUs, and RAM, thereby minimizing energy consumption and device cost. Such improvements enable compact, battery-powered robotic systems, making them more suitable for autonomous mobile platforms.

In short, advancing vision-based grasp detection systems with an emphasis on computational efficiency can significantly impact the field of robotics [4]. It enables robust real-time performance in resource-constrained scenarios and supports broader deployment in practical applications such as logistics, warehouse automation, and smart retail environments.

Mobile manipulators are well-suited for diverse applications [9]; however, research on vision-based mobile manipulators remains limited, particularly in pick-and-pack operations for grocery items [10]. Despite this gap, vision-based manipulation presents a promising direction, with transferable techniques from related tasks such as pick-and-place [11,12], bin-picking [13,14,15], and broader industrial automation applications [16,17]. Interestingly, from the manipulator’s perspective, accurately acquiring object information and determining suitable configurations remain a major challenge in real-world conditions, particularly for object detection and 6D pose estimation, even with learning-based and refinement methods. Existing works tend to prioritize task succession over computational efficiency, often resulting in redundant resource usage. While navigation capabilities are under attention, they are typically well-handled by commercial platforms such as Mobile Industrial Robots (MiRs), which offer high-precision localization and obstacle avoidance. In terms of tasks and operations, autonomous applications require greater emphasis on decision-making strategies and machine vision.

In vision-based robotic systems, various types of sensory input are commonly employed, including RGB images, depth maps, and point clouds. RGB data provide rich visual information for object recognition and classification, while depth and point cloud data offer essential geometric information for identifying object shapes, poses, and spatial locations.

To improve efficiency and reduce computational demands, input data can be optimized. Instead of processing entire scenes, focusing on regions of interest (ROIs) significantly reduces data size and processing time. Otherwise, large point clouds and high-resolution images increase storage requirements and computational complexity. Selecting the appropriate input resolution and limiting the number of point cloud elements can help achieve a balance between system performance and resource efficiency.

A primary component of the perception system is object detection. Traditional methods, such as edge detection, template matching, and feature-based techniques, have been widely used. However, in complex and dynamic environments like grocery shelves, traditional approaches often fail in terms of robustness and adaptability. Modern learning-based methods, which are categorized into one-stage and two-stage detectors, offer superior accuracy and generalization capabilities. Although two-stage methods (e.g., Faster R-CNN) are known for higher accuracy, they require more computational resources and longer inference times. Conversely, one-stage methods (e.g., YOLO, DETR, and SSD) are more suitable for real-time applications due to their speed and relatively lightweight architecture [18].

In systems where low computational costs and a fast response are priorities, such as mobile manipulation in constrained environments, one-stage object detection methods are often preferred. Even if much of the existing literature focuses on maximizing accuracy and task success rates, there is a growing interest in methods that can maintain acceptable performance while reducing computation and power consumption.

Object detection provides object category classification, while segmentation offers precise object boundaries, both critical for effective grasp detection in grocery tasks. To meet the demands of low computational cost, real-time performance, and high accuracy, the combination of YOLO and FAST-SAM has emerged as an effective solution. This pairing forms a foundation for zero-shot detection and precise 3D model reconstruction, as discussed in [19]. However, its performance must be rigorously evaluated in the context of pick-and-pack grocery applications, with opportunities to further optimize for computational efficiency and task-specific requirements.

Object pose estimation presents a similar trade-off [20]. While state-of-the-art pose estimation is largely driven by deep learning, these models often require large, high-quality datasets for training, which can be costly and time-consuming to generate, particularly when targeting specific object types or applications. Moreover, some recent learning-based methods achieve impressive speed and accuracy, but their suitability for deployment on edge devices remains limited owing to computational demands.

In this regard, revisiting traditional geometric approaches can offer practical benefits. These methods rely on straightforward mathematical models and simpler structures, enabling efficient and interpretable pose estimation. For instance, pose estimation directly from object detection outputs, such as bounding box orientation or contour analysis, can yield sufficiently accurate results for many practical applications, without the overhead of complex learning models. Such strategies contribute to building lightweight, real-time robotic systems capable of operating effectively in real-world grocery environments.

Recent advancements in robotic grasp detection have been largely driven by learning-based methods, particularly deep neural networks, because of their high accuracy and real-time performance [21]. These methods, however, often rely on complex architectures that require significant computational resources and access to large and high-quality annotated datasets. Several studies have attempted to reduce model complexity by cutting excessive layers or optimizing network structures to create more lightweight alternatives. While these efforts offer moderate improvements in efficiency, they do not fundamentally reduce the overall computational burden nor present novel methodologies that significantly shift the state of the art.

In this study, we evaluated both the strengths and limitations of learning-based and traditional methods. Deep learning remains crucial for object detection thanks to its superior classification and localization capabilities compared to classical approaches. Alternatively, for object pose estimation and grasp estimation, it is worthwhile to explore and enhance traditional methods. These approaches, when combined with lightweight algorithms, can eliminate the need for resource-intensive processes such as 3D CAD modeling, dataset generation, and matching strategies. This reduction in system complexity could enable faster inference and lower computation cost, an essential requirement for edge computing devices.

A representative state-of-the-art traditional method is presented in [22,23], which performs object-agnostic grasping using 3D point clouds without prior object models. The technique identifies the main object axis and centroid, defines a cutting plane perpendicular to this axis, and generates candidate grasping regions. These candidates are ranked based on estimated grasp stability. The system demonstrated an average processing time of 17.5 ms per object and achieved an 85.55% success rate over 500 grasp attempts with 27 different objects. Exploring thoroughly, it lacks object classification capability, and its segmentation accuracy in cluttered scenes and complex backgrounds is limited. Moreover, the hardware used (Intel i7-4770 CPU (Intel, Santa Clara, CA, USA) at 3.4 GHz with 8 GB of RAM) and stationary manipulator represent a high-performance desktop setup, which is significantly more powerful than edge devices such as NVIDIA Jetson platforms (NVIDIA, Santa Clara, CA, USA).

In contrast to the above, our work is based on a mobile manipulator equipped with an eye-in-hand camera configuration. This setup introduces additional challenges in precision and dynamic positioning, as both the manipulator and its base are mobile. These complexities necessitate the use of computationally efficient methods that can support real-time operation under constrained resources, highlighting the importance of lightweight, optimized algorithms tailored for embedded robotic systems.

In practical robotic applications, obtaining a complete 3D model of an object from point clouds is often challenging due to occlusions and limited sensor coverage. Accurate reconstruction typically requires capturing the object from multiple viewpoints, which significantly increases both processing time and computation. That is the reason why image-based grasp detection methods have gained increasing attention for their efficiency and simplicity. The approach presented in [24] employed a combination of RGB and depth images using a weighted background subtraction method to isolate and segment the object. Principal Component Analysis (PCA) was then applied to determine the object’s primary axis, enabling the generation of candidate grasping rectangles. These candidates were evaluated using a LightGBM classifier to select the most suitable grasp. This method demonstrated notable efficiency, achieving a detection time of only 0.3 ms and an average grasp success rate of 91.81%.

Upon closer examination, the system in [24] was based on an eye-to-hand camera configuration and limited to 2D grasp representations. Furthermore, the experiments were conducted using a high-performance desktop setup (Intel i5-6400 CPU with 16 GB of RAM), which may not be suitable for deployment in mobile robotic platforms. In mobile manipulators equipped with eye-in-hand cameras and constrained by edge computing resources, further optimization of input resolution and algorithmic design is necessary. Leveraging low-resolution RGB inputs and efficient techniques such as PCA offers a promising direction for achieving lightweight, real-time grasp detection suitable for embedded robotic systems.

In addition to image-based grasp detection, analytic grasp stability and quality are critical for overall task success [25]. While physical properties such as weight and surface friction cannot be directly inferred from visual data, form-closure analysis based on object pose estimation provides a practical alternative. In this context, aligning the gripper approach with the object’s centroid and normal surface enhances stability, offering a vision-based approximation of grasp robustness suitable for practical applications.

In summary, the development of a computationally lightweight vision-based grasp detection system for mobile manipulators in pick-and-pack grocery applications requires the careful optimization of input data and system parameters, the implementation of efficient and effective core methodologies, and rigorous validation through real-world experimentation to demonstrate practical applicability.

#### 1.1.2. Benchmarking

To ensure meaningful progress in the development and implementation of robotic systems, performance evaluation is essential. Benchmarks play a critical role in this process by providing standardized metrics that allow for objective comparison across different methods and studies. In robotic grasp detection and manipulation, numerous benchmarks have been proposed for both simulated environments [26,27] and real-world applications [28].

The custom benchmark will address the challenging “Select and Pack” shopping task inspired by the European Robotics League (ERL) competition framework [29]. The experimental setup and validation criteria are aligned with typical robot competition environments. Evaluation metrics and strategies commonly used in robotics competitions such as bin-picking [30] were explored and evaluated to ensure reasonable and achievable validation for grocery-related applications.

The specific application of pick-and-pack operations involving grocery items presents special challenges that are not fully addressed by existing benchmarks. Realistic evaluation in this domain requires consideration of the wide variety of products, shapes, sizes, and packaging types typically found in grocery stores. As this work targets an end-to-end system, all individual modules, such as object detection, segmentation, pose estimation, and grasp planning, must perform reliably to ensure overall task success. A failure in any single module, such as object detection, can lead to a breakdown in the entire process.

Each module can initially be assessed using existing, well-established benchmarks before being integrated into the full system. To avoid redundancy and maintain key metrics, this work only established the evaluation of the whole process of real-world pick-and-pack grocery benchmarks. In evaluating the complete system, the task success rate serves as a primary performance metric, reflecting the proportion of completed pick-and-pack operations. Robustness and reliability can be demonstrated through repeated trials; a higher number of consistent executions indicates improved repeatability and system dependability.

Execution time is another critical metric, particularly in real-time applications. It not only reflects the system’s response but also provides insights into computational efficiency. Lightweight computation is remarkably important for deployment on resource-constrained platforms such as edge devices. Although high-performance computing hardware can boost speed and accuracy, for a fair comparison across studies, it is essential to document processing times along with hardware specifications. Since hardware and software configurations vary depending on budget constraints and application-specific requirements, a direct comparison of computational performance across systems can be challenging. To address this, the compute-to-speed ratio (CSR) is introduced as a meaningful metric for evaluating both computational efficiency and energy consumption. This ratio enables a standardized assessment of system performance, particularly for energy-constrained robotic applications [26].

These performance metrics, namely success rate, robustness, execution time, and hardware configuration, are vital for advancing research in practical, real-world pick-and-pack applications. As the demand for intelligent automation in the service sector continues to rise, developing standardized benchmarks tailored to this domain becomes an important direction for the research community.

This project presents the implementation of an end-to-end, computationally lightweight vision-based grasp detection system designed specifically for pick-and-pack grocery item applications. The primary contributions of this work are summarized as follows:

1. Core Lightweight System Architecture: The essential modules of the proposed grasp detection system were systematically analyzed and defined. Each component, ranging from object detection to grasp estimation, was individually evaluated and optimized to achieve not only computational efficiency but also high success rates in real-world robotic grasping tasks. The overall system architecture was carefully designed, and its core functionalities are presented in detail.
2. Improved Traditional Object Pose Estimation Method: An efficient pose estimation method based on traditional image processing techniques was developed to reduce the complexity and computational demands typically associated with complicated networks. This approach enables precise object pose prediction for unseen objects, without the need for datasets or prior grasping knowledge. Moreover, the method can be seamlessly integrated with various object detection algorithms, enabling broad applicability across different domains.
3. Kinematic Model and Control of a Mobile Manipulator: A complete calculation model of the mobile manipulator system was derived, including the transformation from the camera frame to the robot frame. The robotic arm (UR16e) was mounted on a mobile platform (MiR100), and a unified motion control framework was developed. This framework integrates forward and inverse kinematics to coordinate both joint positioning and end-effector orientation (roll, pitch, and yaw), enabling precise robot operation.
4. Dataset and Benchmark for Grocery Pick-and-Pack Tasks: A dedicated dataset and benchmark for vision-based grasping of grocery items in real-world environments were created and made publicly available. The object detection dataset includes realistic items commonly found in supermarkets. The benchmark framework supports evaluation across diverse robot and gripper configurations and uses standardized metrics such as individual success rate, average success rate, processing time, and the compute-to-speed ratio. These metrics offer a unified and reproducible means of validating system performance and enabling fair comparisons with related works.

## 2. Proposed Methodology

### 2.1. An End-to-End Grasp Detection System

An effective grasp detection system comprises several connected modules, each of which must be carefully evaluated and optimized to ensure high success rates and fast performance in real-world robotic applications. As noted in previous studies [6], vision-based grasping systems are composed of essential building blocks whose configuration may vary depending on the task requirements and operational constraints.

Object detection is a critical first step in the pipeline, enabling the system to classify and localize objects within the image frame. In this project, multiple detection approaches were investigated, including one-stage and two-stage learning-based methods, as well as traditional computer vision techniques. One-stage detectors and traditional methods were found to offer advantages in terms of computational efficiency and processing speed, whereas two-stage and deep learning-based methods typically achieve higher accuracy. Given the constraints of this application, which prioritizes real-time responses on edge devices, a balance was sought, favoring high-speed detection while maintaining sufficient accuracy to ensure task success.

Following object detection, object segmentation is used to extract more detailed object shape information, as bounding boxes alone do not provide sufficient information for precise grasp planning. Regardless, relying solely on instance segmentation has limitations, particularly in terms of classification and localization accuracy. The integration of state-of-the-art object detection (YOLO) with instance segmentation (FAST-SAM) enhances overall system precision, achieves real-time performance, and improves robustness in practical robotic applications [19]. Since the pick-and-pack grocery application requires correct item classification to avoid order errors, the proposed method combines detection and segmentation. The object detector provides a bounding box center that guides instance segmentation to isolate the target object more precisely. Various segmentation algorithms were assessed, with emphasis placed on achieving a lightweight and accurate real-time implementation.

Next, a set of image processing techniques is applied to estimate the object pose, which is essential for determining the appropriate robotic configuration relative to the object’s information. The output from the segmentation module is first converted into a binary image, thereby reducing computational load by eliminating unnecessary color information. Contours are then extracted and analyzed using Principal Component Analysis (PCA), which yields the object’s primary and secondary axes. These axes, along with a derived point cloud representation, are used to estimate the grasping point and the object’s orientation.

**Figure 1:**
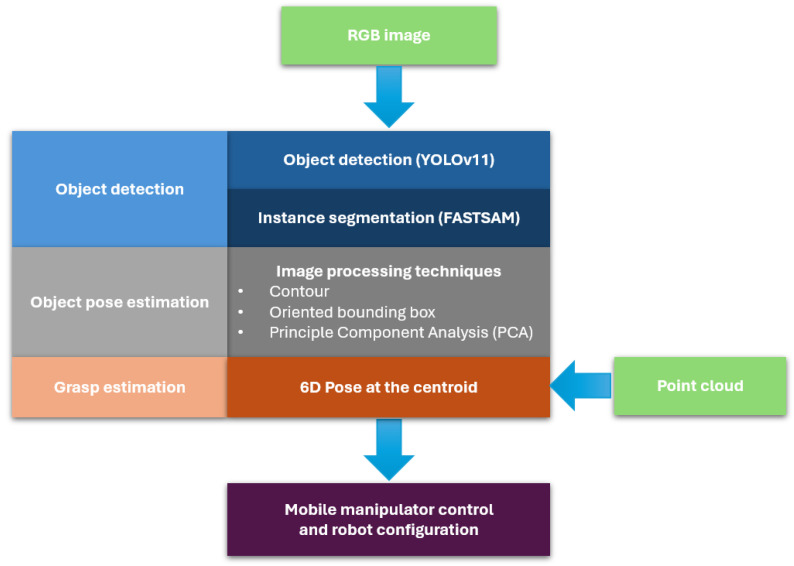
System architecture.

The grasping point is chosen as the centroid of the object’s pixel distribution, offering grasp stability, especially in non-cluttered environments. The complete system architecture and workflow are illustrated in Figure 1, highlighting the integration of detection, segmentation, and pose estimation components.

### 2.2. Comprehensive Core Modules

#### 2.2.1. Object Detection, Yolov11

Object detection is a fundamental task in machine vision, aiming to identify both the class and location of objects within an image. The key performance metrics of any object detection algorithm include classification accuracy, localization precision, and processing speed. Over the past two decades, numerous approaches have been developed, evolving from traditional hand-crafted methods to modern deep learning-based models. In recent years, learning-based techniques have shown remarkable improvements in both accuracy and computational efficiency [18].

Among the state-of-the-art models, the You Only Look Once (YOLO) series has emerged as a leading solution, offering a favorable trade-off between speed and accuracy. After extensive evaluation focusing on fast inference and efficient computation, YOLOv11 was selected for this project. This choice is further supported by the model’s active development community, ease of implementation, and accessibility via public platforms. While alternative algorithms, such as two-stage detectors (e.g., Faster R-CNN), may provide higher accuracy in some scenarios, they typically incur greater computational costs and slower processing times, making them less suitable for real-time mobile manipulation tasks.

In grasp detection, the precise localization of objects in the image frame is critical, as it forms the camera frame for calculating transformations to the robot’s coordinate frame. Although object classification can be valuable, it is not always necessary depending on the task. For example, in applications such as clearing workspaces or picking identical items on a conveyor, object recognition becomes optional. Inevitably, in applications like grocery pick-and-pack, classification is essential to ensure that the correct item is selected based on user-specified orders. In such cases, the robot must not only identify and grasp target items but also navigate to each destination within its environment, requiring coordination from both the manipulator and the mobile base. For that reason, the system should be precise and computationally efficient, factors that reinforce the suitability of YOLOv11.

Turning to the matter of input, the object detection module operates on RGB images as input. Even though multiple input resolutions are supported, they should ideally match the resolution of the training dataset to avoid a loss of accuracy due to automatic resizing. In this study, the camera’s lowest resolution setting (673 × 376 pixels) was selected for input to prioritize real-time performance. The training dataset was configured at 640 × 640 pixels, a resolution that aligns closely with the input. Higher resolutions could improve accuracy but at the cost of increased processing time.

**Figure 2:**
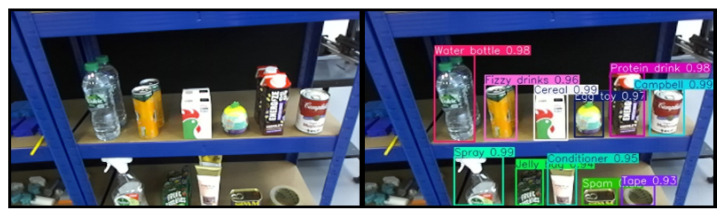
From raw image to object detection (Yolov11).

To enhance generalization and robustness under varying lighting and environmental conditions, several image augmentation techniques were applied during training, including horizontal flipping, brightness adjustment, and cropping. All versions of YOLOv11 were trained on this dataset and evaluated using both test images and real-world scenes. The results demonstrate that the YOLOv11-nano model offers the best balance between speed and model size, with only a slight reduction in accuracy and confidence compared to larger variants. A confidence threshold of 0.7 was defined, providing sufficient reliability for the grocery pick-and-pack task. Based on these findings, YOLOv11-nano was adopted as the object detection model for the proposed system. Figure 2 illustrates the output of the object detection module, where the detected objects are localized within the input image frame using bounding boxes. Each object is assigned a class label along with a corresponding confidence score that reflects the model’s certainty in the classification.

#### 2.2.2. Object Segmentation

Object segmentation involves extracting the shape of an object in the form of contours, which is essential for accurate robotic grasping. Various segmentation models exist, each offering different trade-offs in terms of accuracy, computational cost, and applicability. In this study, particular emphasis is placed on computational efficiency, processing speed, and suitability for real-time grasping tasks in mobile manipulators.

To address these requirements, this work adopts the Fast Segment Anything Model (FAST-SAM) for instance segmentation. FAST-SAM [31] employs YOLOv8-seg as its backbone and leverages region-of-interest (ROI) prompts to extract object masks. This approach enables real-time performance while significantly reducing the computational overhead, all without compromising segmentation accuracy. Importantly, FAST-SAM operates without requiring additional training, as it performs class-agnostic object extraction, distinguishing objects from the background without the need for classification. This functionality is well-suited for grasping tasks, where identifying object boundaries is more critical than class labels.

In the proposed framework, object detection outputs, comprising class labels, bounding box coordinates, and bounding box centers, are used as input prompts for FAST-SAM. The ROI can be specified either by the bounding box coordinates or the center point. Experimental results indicate that using bounding box coordinates yields more precise segmentation compared to using only the center point, as it provides a constrained region that effectively localizes the object.

**Figure 3:**
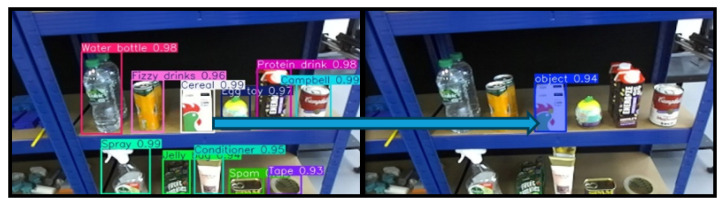
From object detection to instance segmentation (FAST-SAM).

A confidence threshold of 0.6 was selected for the instance segmentation to balance precision and recall in diverse and cluttered environments. Higher confidence thresholds were found to reduce the number of detections in complex scenes. The output of this segmentation stage is a set of object contours, which serve as the foundation for further geometric and pose analysis in the subsequent stages of the grasping pipeline. As illustrated in Figure 3, the target object, a cereal box, is first identified through object detection. The corresponding bounding box is then used as the ROI prompt for segmentation using FAST-SAM, resulting in the extraction of the object’s contour.

#### 2.2.3. Image Processing and Principle Component Analysis (PCA)

**Figure 4:**
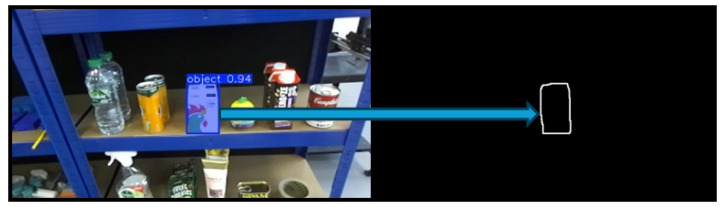
Extracting information from instance segmentation by FindContour and DrawContour.

After instance segmentation, image processing techniques from the OpenCV library are applied. First, a binary image of the same dimensions as the input image is generated. Contour detection is then performed on the segmented output, and the identified contours are drawn onto the binary image. This process is illustrated in Figure 4.

**Figure 5:**
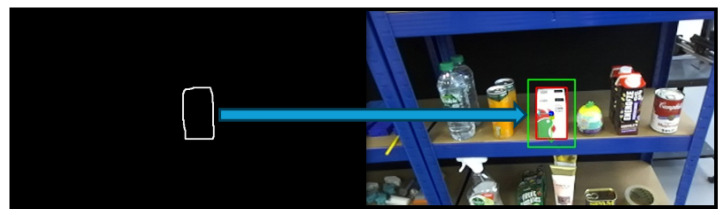
Performing PCA and fitting bounding boxes for object information.

Principal Component Analysis (PCA) is a statistical technique used for linear dimensionality reduction by transforming data into a new coordinate system, where the axes are ordered by the amount of variance they capture. This approach is particularly useful in identifying the main axis of an object within a digital image, which aids in estimating orientations and optimal grasping points. In this work, once the target object is segmented, its contour is extracted and converted into a binary image. This conversion reduces the data from RGB color channels (red, green, and blue) to two values (black and white), thus lowering computational complexity. PCA is then applied to the set of data points within the contour to compute the centroid (mean position), the primary axis (direction of maximum variance), and the secondary axis. In addition, an enhanced reference point is generated along the primary axis (adjacent to the centroid) to assist in determining the orientation of the object. This point serves as a directional indicator for grasp alignment. Subsequently, an oriented bounding box is computed to tightly fit the object’s contour using the MinAreaRect function from the OpenCV library. Furthermore, an enhanced bounding box is constructed based on the object orientation and gripper dimensions to evaluate the surrounding space for potential collisions. This extended bounding box helps assess whether the gripper can approach and engage the object without interference. The process is illustrated in Figure 5.

After extracting the object’s contour, the set of pixel coordinates (x,y) within the object boundary is used to compute Principal Component Analysis (PCA) as described in the equations below, enabling estimation of the object’s centroid and principal orientation.

PCA for object pose estimation

Given a 2D object’s contour represented by *N* points $\left(x_{i},y_{i}\right)\in R^{2}$, where i=1,2,…,N, PCA can be used to estimate the object’s orientation.

Centroid Calculation:

First, compute the centroid of the contour points as follows:

(1) $$\bar{x}=\frac{1}{N}\sum_{i=1}^{N}x_{i},\;\bar{y}=\frac{1}{N}\sum_{i=1}^{N}y_{i}$$

(2) $$\bar{p}=\left[\begin{array}{c} \bar{x}\\ \bar{y} \end{array}\right]$$

Covariance Matrix:

Center each point by subtracting the centroid as follows:

(3) $$p_{i}=\left[\begin{array}{c} x_{i}-\bar{x}\\ y_{i}-\bar{y} \end{array}\right]$$

Then construct the covariance matrix as:

(4) $$C=\frac{1}{N}\sum_{i=1}^{N}p_{i}p_{i}^{⊤}=\left[\begin{array}{cc} \sigma_{xx} & \sigma_{xy}\\ \sigma_{yx} & \sigma_{yy} \end{array}\right]$$

where the elements are:

(5) $$\begin{array}{cc} \sigma_{xx} & =\frac{1}{N}\sum_{i=1}^{N}{\left(x_{i}-\bar{x}\right)}^{2} \end{array}$$

(6) $$\begin{array}{cc} \sigma_{yy} & =\frac{1}{N}\sum_{i=1}^{N}{\left(y_{i}-\bar{y}\right)}^{2} \end{array}$$

(7) $$\begin{array}{cc} \sigma_{xy} & =\sigma_{yx}=\frac{1}{N}\sum_{i=1}^{N}\left(x_{i}-\bar{x}\right)\left(y_{i}-\bar{y}\right) \end{array}$$

Eigen Decomposition:

To find the object’s principal orientation, perform eigen decomposition on the covariance matrix as follows:

(8) $$Cv_{k}=\lambda_{k}v_{k}$$

where $\lambda_{k}$ represents the eigenvalues and $v_{k}$ represents the corresponding eigenvectors. The eigenvector with the largest eigenvalue represents the object’s principal axis.

Orientation Angle:

The object’s orientation angle (roll in 2D) is given by:

(9) $$\theta =\arctan2(v_{1y},v_{1x})$$

where $v_{1}=\left[\begin{array}{c} v_{1x}\\ v_{1y} \end{array}\right]$ is the eigenvector associated with the largest eigenvalue.

**Figure 6:**
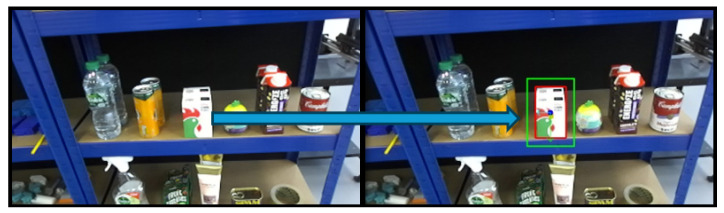
From the raw image to object pose estimation result and the grasping point.

The centroid approximates the geometric center of the object, while the primary axis represents its dominant orientation. These features provide a reliable basis for aligning grasping points along the primary axis to improve grasp stability. The centroid is generally considered a favorable grasping point due to its balanced position and symmetry, while the orientation angle is for roll control. Based on this approach, the input RGB image is processed to estimate both the object’s pose and suitable grasping points. This process is illustrated in Figure 6.

However, grasping points may need to be adjusted based on the object’s geometry and environmental constraints. For example, the object’s width at the centroid may exceed the gripper’s span, or surrounding obstacles may obstruct the grasp approach. Such factors can significantly impact the success rate of grasping actions. Therefore, incorporating alternative grasp points and a decision-making mechanism becomes essential for robust grasp planning in real-world applications.

The integration of optimally selected inputs and lightweight methods results in a total system model size of 30.9 MB, excluding standard packages such as OpenCV, ur-rtde, and the ZED SDK. The system is designed for real-time performance and deployment in real-world applications. While the methodology plays a critical role, precise robot control and motion planning are equally essential to ensure reliable task execution.

### 2.3. Fallback Strategy

To ensure robustness and practical applicability, a fallback strategy was implemented to address potential failures in the perception and grasping pipeline. The primary RGB input is acquired from the ZED2i stereo camera’s left sensor; if unavailable, the system automatically switches to the right sensor with an updated transformation matrix. If neither camera is operational, the system halts for safety and flags on-site maintenance, which may be required due to hardware faults or USB disconnections.

If object detection or pose estimation fails, the robot repositions to acquire an alternative viewpoint. Persistent failure triggers a report indicating either the absence of the target item or an unreachable configuration, which is in compliance with safety constraints.

**Figure 7:**
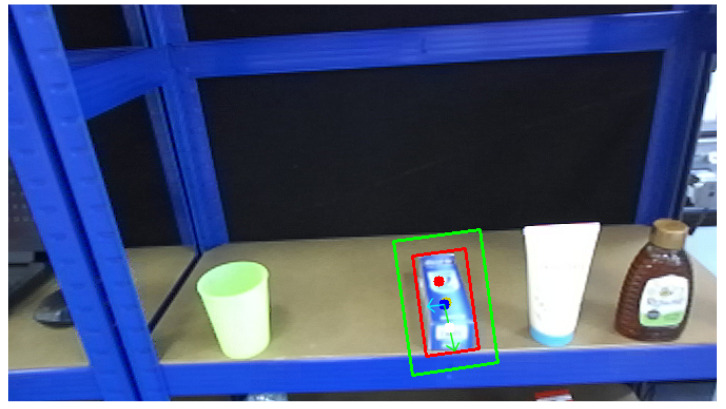
PCA ambiguity in angle prediction, while the red dot is enhanced point from PCA to calculate aspect ratio together with the blue dot, which is centroid point. The arrows show primary and secondary axis.

Pose estimation is performed using Principal Component Analysis (PCA) to extract the object’s main axis and roll angle. Perspective distortion may induce minor angular deviations; for example, a vertically aligned toothpaste tube was misestimated by approximately $5^{\circ }$, as shown in Figure 7. To mitigate this, offset calibration is applied. Following the tolerance thresholds reported in the Cornell [26] and Jacquard [27] datasets, roll angles within $\pm 15^{\circ }$ are considered acceptable. After calibration, the roll error in the example case was reduced to ≤$5^{\circ }$, enabling a successful grasp.

The robot operates within designed work envelope limits to ensure safe and feasible motion. For example, the *X*-coordinate must remain within [0.40,0.75] m; if any of the *X*, *Y*, or *Z* positions fall outside their respective ranges, the system reports “no possible solutions” and aborts the motion. Such cases are rare due to the mobile platform’s ability to position the manipulator at an appropriate distance from the target.

The manipulator is equipped with joint-level force sensing, enabling an immediate emergency stop upon contact with any obstacle, prioritizing operator and system safety. Furthermore, if any values (e.g., camera coordinates and calculations) fall outside the permissible range, the system halts and flags the fault for precise correction. If the fault persists after an automated retry, it is classified as a critical error requiring human intervention.

This design philosophy ensures that the system operates autonomously under normal conditions while respecting safety constraints inherent to collaborative robot tasks.

### 2.4. The Design and Calculation Model of a Mobile Manipulator

**Figure 8:**
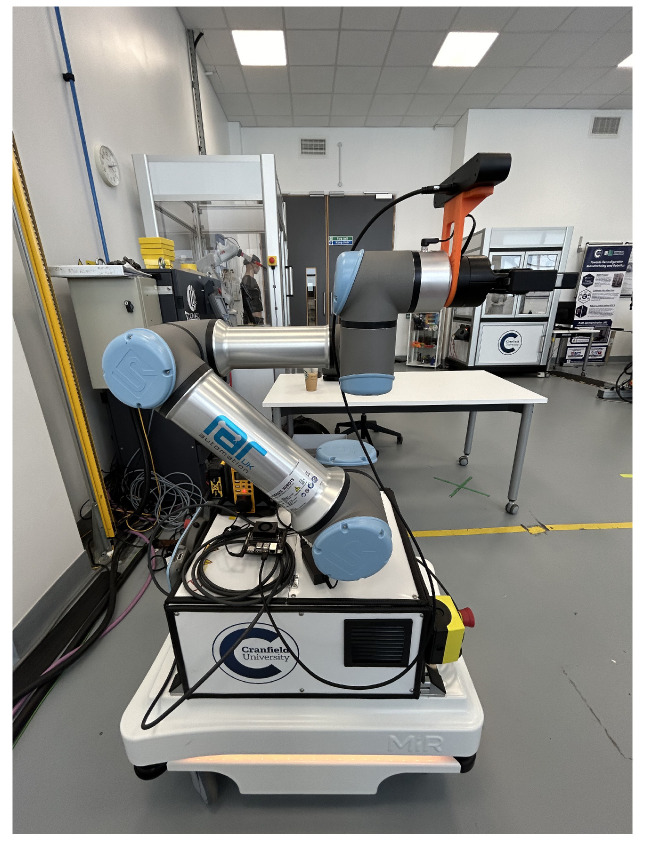
Mobile manipulator (UR16e and MiR100).

Each robotic system features specific dimensions, configurations, and camera mounting strategies that must be considered for accurate manipulation. In this project, the camera is mounted on the end effector in an eye-in-hand configuration, while a Universal Robots (UR16e) robotic arm is installed on a Mobile Industrial Robots (MiR100) platform, as illustrated in Figure 8.

The selection of an appropriate camera is critical for achieving accurate and reliable grasp detection in robotic applications. A wide range of industrial cameras is available on the market, including low-cost webcams, RGB and RGB-D cameras, and stereo vision systems, many of which are commonly used in robotic grasping research. In this project, the ZED2i stereo camera was selected as a cost-effective yet high-performance solution. The ZED2i provides robust depth sensing and high accuracy across a range of environmental and lighting conditions, making it particularly well-suited for real-world deployment [32].

Following the selection of the camera, an appropriate edge computing device was evaluated to ensure real-time processing capability and hardware compatibility. The NVIDIA Jetson Orin Nano was identified as a high-performance edge computing platform featuring powerful GPU capabilities, making it suitable for computationally intensive tasks such as vision-based grasp detection. Importantly, it also meets the minimum hardware requirements of the ZED2i stereo camera, which relies on GPU acceleration for depth processing and visual data acquisition. Compatibility between the edge device and the camera is crucial, as certain ZED SDK functions require a minimum level of CPU and GPU performance. Failure to meet these requirements may result in technical issues or software incompatibilities that hinder system performance.

The Jetson Orin Nano was connected to the ZED2i camera via a USB interface. The UR16e robotic arm was networked with the MiR100 mobile platform through a wired local area network (LAN) connection, ensuring stable and fast communication. Meanwhile, the Jetson Orin Nano was connected to both the MiR100 and the local network via Wi-Fi. This configuration allowed a laptop to remotely access and control the edge device over the local network using a remote desktop interface.

**Figure 9:**
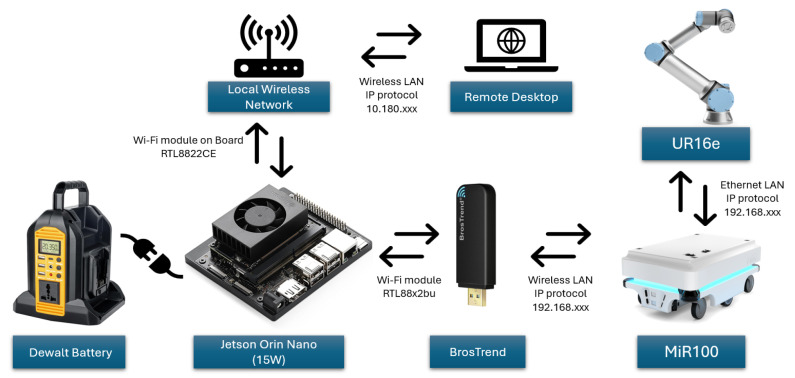
Hardware connection.

Although Wi-Fi communication is generally slower than wired connections, it offers greater flexibility for system deployment by enabling wireless control and unobstructed robot mobility. This design avoids cable entanglement and supports seamless operation in dynamic environments. An overview of the hardware connectivity architecture is illustrated in Figure 9.

As the grasping task in this study relies heavily on visual perception, the accuracy of the spatial information, specifically object coordinates and distance measurements, directly affects the success of manipulation. Any error in visual input may propagate to the robot’s motion planning, potentially resulting in grasp failures. Therefore, it is crucial that the camera’s coordinate system be precisely calibrated and aligned with the robot frame. The physical mounting of the camera on the robot’s end effector is also incorporated into the transformation matrix calculations, ensuring accurate pose estimation and alignment in the robot’s coordinate space.

**Figure 10:**
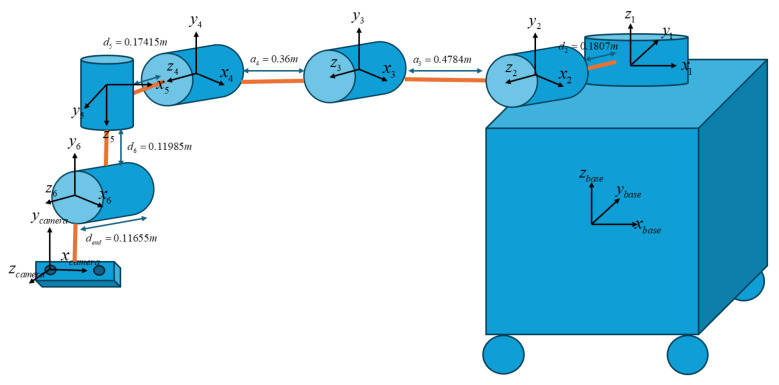
Initial pose (UR16e).

Accurate transformation among the object frame, image frame, camera frame, and robot base frame is essential for achieving precise manipulation. To establish these transformations, the physical dimensions of all robot joints and the camera mount must be measured carefully. It is critical to initialize the manipulator in a zero position, where all joint angles are set to zero, following Figure 10. Forward kinematics can then be formulated using the Denavit–Hartenberg (DH) convention, as detailed in Table 1, to determine the position and orientation of the tool center point (TCP).

Under this configuration, each transformation matrix $T_{i}^{i-1}$ represents the coordinate transformation from frame {i} to frame {i−1}. Based on these transformations, the camera frame can be accurately linked to the robot base frame through a defined kinematic chain, allowing the object coordinates obtained from the camera to be expressed in the robot’s workspace.

(10) $$T_{i}^{i-1}=R_{X}\left(\alpha_{i-1}\right)\;D_{X}\left(a_{i-1}\right)\;R_{Z}\left(\theta_{i}\right)\;D_{Z}\left(d_{i}\right)$$

(11) $$\begin{array}{c} T_{0}^{\mathrm{base}}=\left[\begin{array}{cccc} 1 & 0 & 0 & 0.085\\ 0 & 1 & 0 & 0\\ 0 & 0 & 1 & 0.310\\ 0 & 0 & 0 & 1 \end{array}\right],T_{1}^{0}=\left[\begin{array}{cccc} \cos(\theta_{1}) & -\sin(\theta_{1}) & 0 & 0\\ \sin(\theta_{1}) & \cos(\theta_{1}) & 0 & 0\\ 0 & 0 & 1 & 0\\ 0 & 0 & 0 & 1 \end{array}\right],\;\\ T_{2}^{1}=\left[\begin{array}{cccc} \cos(\theta_{2}) & -\sin(\theta_{2}) & 0 & 0\\ 0 & 0 & -1 & 0\\ \sin(\theta_{2}) & \cos(\theta_{2}) & 0 & 0.1807\\ 0 & 0 & 0 & 1 \end{array}\right],T_{3}^{2}=\left[\begin{array}{cccc} \cos(\theta_{3}) & -\sin(\theta_{3}) & 0 & -0.4784\\ \sin(\theta_{3}) & \cos(\theta_{3}) & 0 & 0\\ 0 & 0 & 1 & 0\\ 0 & 0 & 0 & 1 \end{array}\right],\;\\ T_{4}^{3}=\left[\begin{array}{cccc} \cos(\theta_{4}) & -\sin(\theta_{4}) & 0 & -0.36\\ \sin(\theta_{4}) & \cos(\theta_{4}) & 0 & 0\\ 0 & 0 & 1 & 0\\ 0 & 0 & 0 & 1 \end{array}\right],T_{5}^{4}=\left[\begin{array}{cccc} \cos(\theta_{5}) & -\sin(\theta_{5}) & 0 & 0\\ 0 & 0 & -1 & 0\\ \sin(\theta_{5}) & \cos(\theta_{5}) & 0 & 0.17415\\ 0 & 0 & 0 & 1 \end{array}\right],\;\\ T_{6}^{5}=\left[\begin{array}{cccc} \cos(\theta_{6}) & -\sin(\theta_{6}) & 0 & 0\\ 0 & 0 & 1 & 0\\ -\sin(\theta_{6}) & -\cos(\theta_{6}) & 0 & 0.11985\\ 0 & 0 & 0 & 1 \end{array}\right],T_{\mathrm{end}-\mathrm{effector}}^{6}=\left[\begin{array}{cccc} 1 & 0 & 0 & 0\\ 0 & 1 & 0 & 0\\ 0 & 0 & 1 & 0.11655\\ 0 & 0 & 0 & 1 \end{array}\right],\;\\ T_{\mathrm{camera}}^{\mathrm{end}-\mathrm{effector}}=\left[\begin{array}{cccc} 1 & 0 & 0 & -0.0635\\ 0 & 1 & 0 & -0.130\\ 0 & 0 & 1 & 0.030\\ 0 & 0 & 0 & 1 \end{array}\right] \end{array}$$

(12) $$T_{camera}^{base}=T_{0}^{base}\cdot T_{1}^{0}\cdot T_{2}^{1}\cdot T_{3}^{2}\cdot T_{4}^{3}\cdot T_{5}^{4}\cdot T_{6}^{5}\cdot T_{end-effector}^{6}\cdot T_{camera}^{end-effector}$$

Therefore, the derived homogeneous transformation matrix represents the forward kinematics model of the system. The rotation matrix R defines the orientation, while the position vector specifies the translational components *X*, *Y*, and *Z*. Given known joint angles $\theta_{1}$ to $\theta_{6}$, the complete transformation matrix (Equation (13)) can be computed to determine the end-effector pose relative to the robot base.

(13) $$T=\left[\begin{array}{cccc} R_{11} & R_{12} & R_{13} & X\\ R_{21} & R_{22} & R_{23} & Y\\ R_{31} & R_{32} & R_{33} & Z\\ 0 & 0 & 0 & 1 \end{array}\right]$$

**Figure 11:**
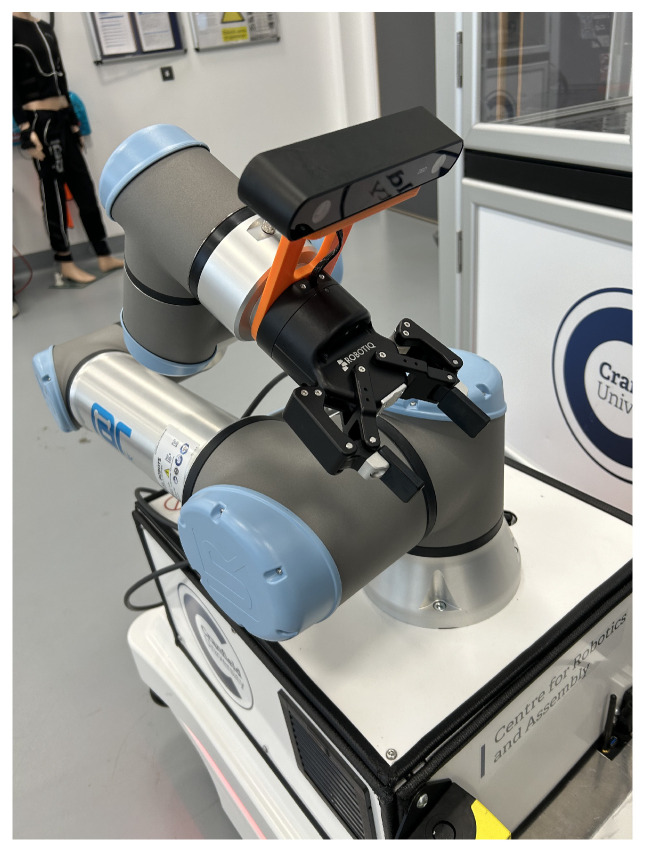
Robotiq 2F-85.

Based on the derived transformation matrices, the system obtains object positions through vision-based detection in the image frame. These pixel coordinates are mapped to 3D point cloud positions in the camera frame, which are then transformed to the end-effector and robot base frames for precise task execution. The grasping strategy employs a parallel gripper (Figure 11) to perform pick-and-pack operations. Regarding the gripper mechanism, the parallel gripper used in this work is capable of controlling both width (0–8.5 cm) and force (20–185 N) with continuous adjustment from 0% to 100%. In the present experiments, the gripping force was fixed at 20 N, while the gripper width was automatically closed until it reached the force limit corresponding to the object’s dimensions. This configuration was validated as sufficient for rigid objects; however, further improvements are required to enable reliable grasping of deformable items, where adaptive force control and compliant strategies become essential. To ensure grasp stability, the system targets the object’s centroid, which offers optimal balance. Accurate centroid detection requires full object visibility without occlusions and a camera viewpoint approximately parallel to the object surface, resembling a 2D grasp.

In this setup, the camera is fixed at a 25-degree angle, determined through empirical testing to provide optimal visibility of both upright and lay-flat objects while simplifying calculations by fixing one joint. A key robustness feature of the system lies in its active alignment: once the point cloud is acquired, forward kinematics is used to transform coordinates to the mobile platform, which then adjusts its X and Y position to align the camera centrally and at a designed distance from the target object. This ensures consistent approach conditions, distance, orientation, and viewpoint, during grasp execution, staying within the robot’s work envelope and avoiding unreachable configurations or singularities.

**Figure 12:**
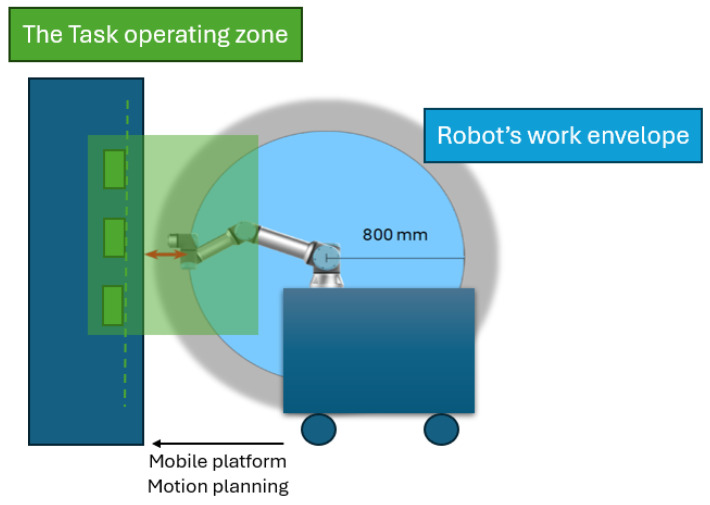
Work envelope.

Moreover, given the manipulator’s limited workspace (work envelope), it may be necessary to reposition the mobile platform to maintain an appropriate distance for manipulation once the target object is detected. A strategy for coordinated mobile base and manipulator positioning can be designed accordingly, as illustrated in Figure 12.

Given a predefined working envelope, inverse kinematics (IK) can be employed to compute the joint angles required to position the robotic manipulator for a desired end-effector pose. Once the forward kinematics of the system is established, the position and orientation of the object relative to the robot base frame can be determined. Subsequently, inverse kinematics is used to solve for the joint configurations necessary to reach the target pose.

In this work, the UR controller [33] utilizes an analytical approach and q-near to compute IK solutions. This solver is selected due to its ability to generate multiple feasible solutions with efficient real-time performance. Among the candidate solutions, the one closest to the current joint configuration is selected to minimize motion deviation and optimize the trajectory. Additionally, task-specific constraints derived from the grasp detection module are integrated into the IK computation to ensure collision-free and task-aware motion planning.

However, the UR controller basically requires the waypoint, and then it will provide the target joints, but it generates multiple solutions that need the conditions to evaluate the proper solution; otherwise the solution will be close to the current pose. To control the robot in advance, the calculation model needs to be derived specifically. This project also derived orientation control for the manipulator such as roll, pitch, and yaw.

Yaw Control: Rotational Matrix Around the Z-axis:

(14) $$R_{z}\left(\theta \right)=\left[\begin{array}{ccc} \cos\theta & -\sin\theta & 0\\ \sin\theta & \cos\theta & 0\\ 0 & 0 & 1 \end{array}\right]$$

Pitch Control: Rotational Matrix Around the Y-axis:

(15) $$R_{y}\left(\theta \right)=\left[\begin{array}{ccc} \cos\theta & 0 & \sin\theta \\ 0 & 1 & 0\\ -\sin\theta & 0 & \cos\theta \end{array}\right]$$

Roll Control: Rotational Matrix Around the X-axis:

(16) $$R_{x}\left(\theta \right)=\left[\begin{array}{ccc} 1 & 0 & 0\\ 0 & \cos\theta & -\sin\theta \\ 0 & \sin\theta & \cos\theta \end{array}\right]$$

The robot obtains its current orientation in the form of a rotation vector, which must be converted into a rotation matrix for subsequent calculations.

Robot Rotational Vector to Rotational Matrix:

(17) $$\left[\begin{array}{c} r_{x}\\ r_{y}\\ r_{z} \end{array}\right]\to \left[\begin{array}{ccc} R_{xx} & R_{xy} & R_{xz}\\ R_{yx} & R_{yy} & R_{yz}\\ R_{zx} & R_{zy} & R_{zz} \end{array}\right]$$

Let $\theta =∥\vec{r}∥$ and unit vector $u=\frac{\vec{r}}{∥\vec{r}∥}$. Then,

(18) $${\left[u\right]}_{\times }=\left[\begin{array}{ccc} 0 & -u_{z} & u_{y}\\ u_{z} & 0 & -u_{x}\\ -u_{y} & u_{x} & 0 \end{array}\right]$$

Let *I* be the identity matrix. Then the rotation matrix is

(19) $$R=I+\sin\theta {\left[u\right]}_{\times }+\left(1-\cos\theta \right){\left[u\right]}_{\times }^{2}$$

Composed Rotation:

(20) $$R_{\mathrm{orientation}}=R_{\mathrm{robot}\_\mathrm{pose}}\cdot R_{\mathrm{rotational}\_\mathrm{around}\_\mathrm{axis}}$$

On the other hand, the rotation matrix can be converted into a rotation vector as shown in Equation (22), where

$$\left[\begin{array}{ccc} R_{xx} & R_{xy} & R_{xz}\\ R_{yx} & R_{yy} & R_{yz}\\ R_{zx} & R_{zy} & R_{zz} \end{array}\right]\to \left[\begin{array}{c} r_{x}\\ r_{y}\\ r_{z} \end{array}\right]$$

Rotation Matrix to Axis–Angle Vector:

(21) $$\theta =\cos^{-1}\left(\frac{\mathrm{trace}(R)-1}{2}\right)$$

(22) $$\vec{r}=\frac{\theta }{2\sin\theta }\left[\begin{array}{c} R_{zy}-R_{yz}\\ R_{xz}-R_{zx}\\ R_{yx}-R_{xy} \end{array}\right]$$

**Figure 13:**
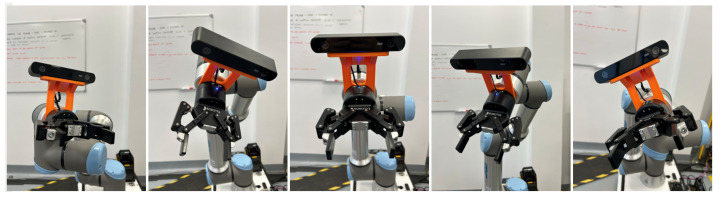
Robot orientation Control.

The final command to the robot will be:

$$\left[\begin{array}{c} X,Y,Z,r_{x},r_{y},r_{z} \end{array}\right]$$

where X,Y,Z represent the target waypoint coordinates obtained from the point cloud, followed by the rotational vector $\left[r_{x},r_{y},r_{z}\right]$ that defines the desired orientation of the end effector in axis–angle form (Figure 13).

At this stage, the robot can be fully controlled to reach target positions and align its orientation relative to the object’s surface. Grasping at the centroid and maintaining perpendicular alignment to the surface normal enhance grasp stability. In this study, the object poses are limited to upright and laydown configurations, corresponding to surface normals of [0,0,1] and [0,1,0], respectively, with pitch control.

This approach demonstrates how the kinematic model for a mobile manipulator can be designed and derived to enable accurate, efficient, and robust performance in pick-and-pack grocery tasks.

### 2.5. Benchmark Methodology

Benchmarking is essential for evaluating the effectiveness of grasping systems and facilitating meaningful comparisons across studies. A robust benchmark requires clearly defined performance metrics, justified object selection, and well-structured experimental setups to ensure consistency, reproducibility, and relevance for future research. In the field of robotic grasping, two principal representation methods are widely used:

2D Rectangle Representation, which is supported by well-known benchmarks such as the Cornell [26] and Jacquard datasets [27];

6D Pose Representation, as exemplified by the GraspNet-1Billion dataset [28].

These benchmarks are primarily based on simulations and are designed to emulate real-world visual perception. The datasets typically include common household items and assume manipulation using parallel-jaw grippers, accompanied by established evaluation metrics. However, for applications such as pick-and-pack grocery tasks, there remains a need for a domain-specific benchmark. These tasks require the system to correctly identify, localize, and grasp specific items in realistic environments.

First, object detection is a crucial module in this application, as the system must accurately classify and localize the target item within the image frame. The number and diversity of objects in the dataset should reflect actual supermarket conditions while excluding irrelevant items to optimize efficiency.

Second, discrepancies between simulated and real-world environments can significantly impact performance. A system that performs well in simulation may not necessarily generalize to physical settings due to differences in lighting, object texture, background complexity, and hardware configurations. Additionally, robot and gripper designs vary across studies, making it difficult to evaluate grasping systems uniformly. For instance, five-fingered robotic hands may be incompatible with benchmarks designed for two-fingered grippers.

To overcome these challenges, the following evaluation metrics are proposed for this benchmark:

Success Rate: A successful grasp is defined as the robot autonomously detecting, grasping, and delivering the correct item from the shelf to a designated delivery area. This metric allows for evaluation across different grippers and robot models. The average success rate over multiple trials reflects both reliability and repeatability.

Execution Time: It is measured from the moment the input image is captured until the grasping point is generated. This excludes robot motion and focuses on computational performance.

Compute-to-Speed Ratio: To assess the energy efficiency of the system, it is defined as the ratio of the frame rate per second (FPS) to the power consumption in watts (W). This metric quantifies the number of image frames the system can process per unit of electrical power consumed, which is crucial for mobile robotic platforms operating under limited energy resources.

(23) $$\mathrm{Compute}-\mathrm{to}-\mathrm{Speed}\;\mathrm{Ratio}\;\left(\mathrm{CSR}\right)=\frac{\mathrm{FPS}}{\mathrm{Power}\;\left(W\right)}$$

A higher CSR value indicates improved energy efficiency, reflecting a better trade-off between computational throughput and energy usage.

Hardware Specification: Detailed system specifications (CPU, GPU, and RAM) must be reported. This facilitates a fair comparison across studies by accounting for computation time, device capabilities, and energy usage.

**Figure 14:**
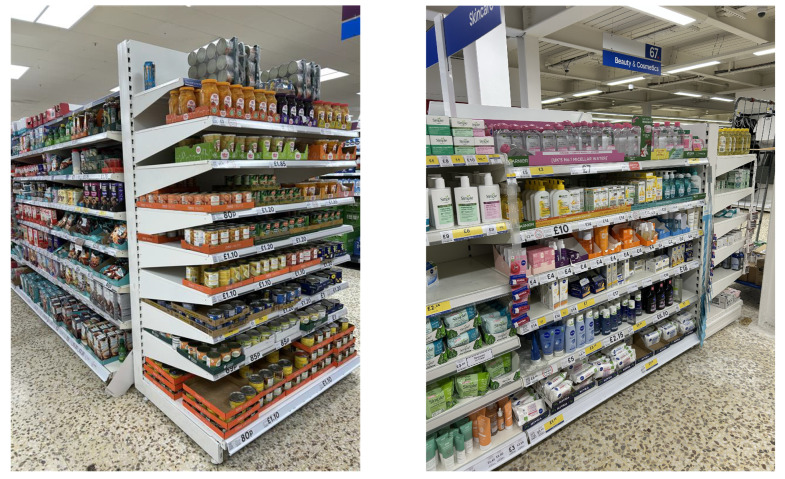
Real-world grocery shopping.

In terms of exploring supermarket environments, as shown in Figure 14, and the nature of grocery items, most of the items are in containers, which are geometric shapes that are well-arranged on the shelves. Meanwhile, some items are of complicated shapes due to their functionality, which is challenging for grasping algorithms.

This benchmark provides an application-driven, real-world evaluation framework for grocery pick-and-pack scenarios. It complements existing grasping datasets by offering task-specific metrics and promoting reproducibility across robotic platforms. To evaluate the proposed system under realistic conditions, a custom benchmark for pick-and-pack grocery tasks was developed. This section outlines the setup, the evaluation protocol, and the three-tier benchmark levels based on increasing task complexity.

The experimental setup consists of a stationary shelf placed on a flat surface, with a mobile manipulator (UR16e mounted on a MiR100 platform) positioned such that both the camera view and the manipulator’s reachable workspace are appropriately aligned. The mobile platform may reposition itself during execution, depending on the grasping configuration, although this behavior is optional and left to the system design.

The benchmark begins by selecting a difficulty level and arranging a predefined set of objects on the shelf. To facilitate unobstructed grasping, a minimum spacing of 5 cm is maintained between items. The delivery area is located at least 3 m away from the shelf, allowing sufficient space for the robot to transport objects.

The system is evaluated based on both computational and operational performance. The execution time is measured from the moment an image is captured to the point at which a grasping position is generated. While the total motion time (including robot travel) may be recorded, it is excluded from benchmark comparisons due to hardware-specific differences (e.g., velocity and acceleration settings).

Hardware specifications, such as the camera type, processing unit (CPU, GPU, and RAM), and robotic platform, must be reported to ensure fair comparisons across systems.

Each benchmark trial requires the robot to autonomously clear all items from the shelf and place them in the delivery area. Items may be repositioned between rounds to evaluate the system’s adaptability to varying object configurations and placements. A grasp is considered successful if the robot correctly detects the object, executes the grasp, and delivers the item to the target zone without dropping or misplacing it. Failures include incorrect detections, failed grasps, or misplacement outside the delivery area.

Each object is tested over five independent trials to ensure statistical reliability. Success rates are recorded per item and averaged across rounds to assess system robustness and repeatability.

The benchmark is divided into three levels of increasing complexity:

Level 1: Basic Geometry and Upright Pose

**Figure 15:**
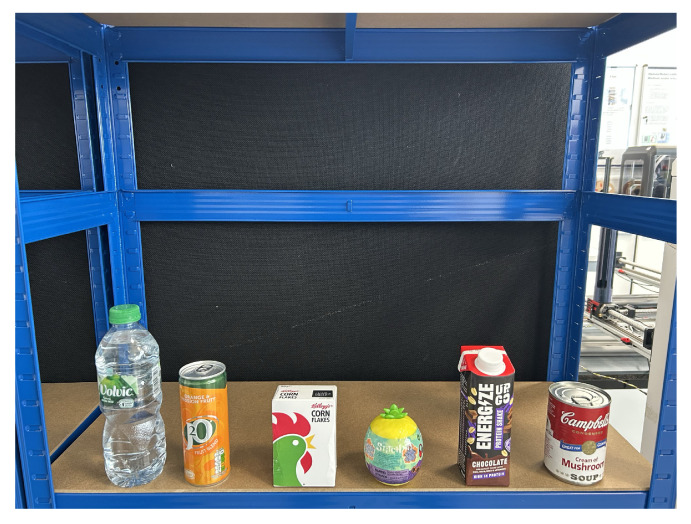
Level 1 benchmark.

This level includes objects with simple geometric shapes and upright poses, such as a water bottles, soda cans, cereal boxes, egg-shaped toys, protein drinks, and soup cans. All objects have widths within 8.5 cm, making them compatible with standard parallel grippers. As objects are uniformly upright and face the camera directly, the task closely resembles a 2D grasping scenario. This level focuses on object detection, basic pose estimation, and straightforward grasp planning (Figure 15).

Level 2: Mixed Poses and Complex Shapes

**Figure 16:**
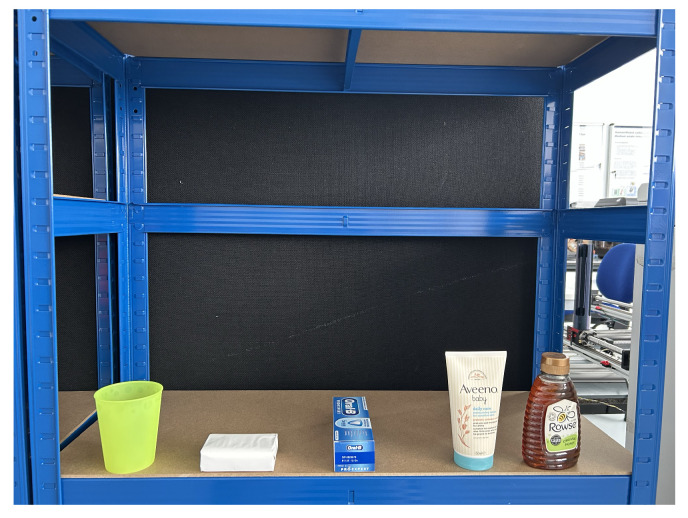
Level 2 benchmark.

This level includes more varied objects such as a glasses, soap bars, toothpaste boxes, lotions, and honey bottles. Items are presented in both upright and horizontal orientations. Due to increased shape complexity, the system must estimate the object’s pose and control the manipulator’s configuration, including orientation adjustments for roll, pitch, and yaw. This level evaluates the system’s ability to handle variation in shape, pose, and grasp location (Figure 16).

Level 3: Grasp Constraints and Affordance Awareness

**Figure 17:**
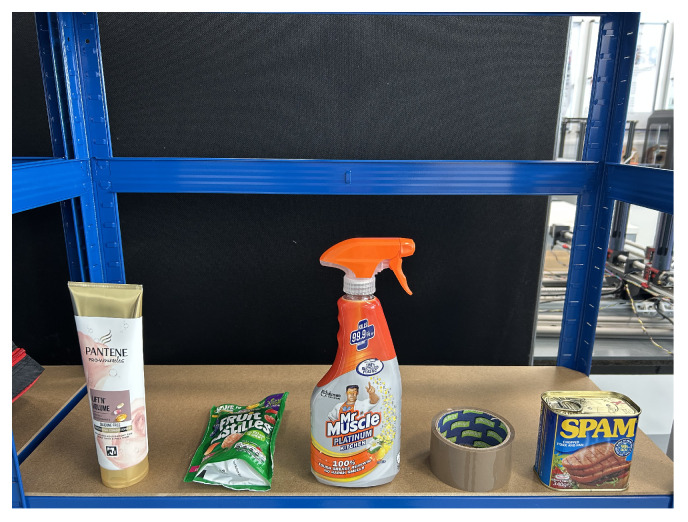
Level 3 benchmark.

This level presents the most challenging scenarios, including items such as a conditioner bottles, sweet bags, cleaner sprays, tapes, and canned meats (e.g., Spam). These objects may exceed the gripper’s width at their centers, include hollow or irregular shapes or exhibit limited graspable surfaces. The system must identify alternative grasping points based on geometry and affordance constraints. Grasp planning must consider surface accessibility and physical limitations (Figure 17).

The experimental setup began with the robot positioned approximately 1.5 m in front of the shelf. The operator selected the target item via the interface, after which the system initiated the perception pipeline, including object detection, pose estimation, and grasp point generation. Processing time was measured from image acquisition to the delivery of the 6D object pose to the robot.

Upon receiving the 6D information, the manipulator configured its pose to grasp the item, and it is supported by the mobile platform to ensure proper reachability. After a successful grasp, the robot navigated 3 m to a delivery table, where the item was placed according to predefined delivery poses aligned with the table dimensions. Each successful placement was counted as one completed trial.

The robot then returned to the starting position to repeat the process until all items were cleared from the shelf. For robustness evaluation, five independent trials were conducted, and average values of key performance metrics were reported.

## 3. Experiments and Results

The experimental setup utilizes a mobile manipulator composed of a UR16e robotic arm (Universal Robots, Odense, Denmark) mounted on a MiR100 mobile platform (Mobile Industrial Robots, Odense, Denmark). A ZED 2i stereo camera (Stereolabs, San Francisco, CA, USA) is mounted in an eye-in-hand configuration at the end effector to provide visual input. The system is powered by a Jetson Orin Nano, featuring a 6-core Arm^®^ Cortex^®^-A78AE v8.2 64-bit CPU (1.5 MB L2 + 4 MB L3), a 1024-core NVIDIA Ampere architecture GPU with 32 Tensor Cores, and 8 GB of RAM. The processing unit is connected to a laptop via remote desktop over a local wireless network, primarily for remote robot control and system monitoring.

For safety and consistent performance, the UR16e robotic arm was configured with a velocity of 0.25m/s and an acceleration of $0.50\;m/s^{2}$. Since robot movement duration is highly dependent on these parameters, it is excluded from the measured execution time. Instead, the focus is placed solely on processing time to evaluate the responsiveness and computational efficiency of the proposed system, which is the primary objective of this methodological development.

In accordance with the pick-and-pack benchmark protocol, each item undergoes five grasping attempts. To ensure robustness and context-aware analysis, the specifications of the processing device are documented prior to experimentation. The execution time is measured from the moment the system begins capturing images to the point at which grasping point coordinates are generated in Cartesian space. In this project, the time module in Python version 3.10.7 was employed to record the start and end times of the processing cycle.

A successful grasp is defined as the robot accurately retrieving the item from the shelf and depositing it in the designated delivery area. This process encompasses the complete pipeline, from visual detection to physical grasp execution. System reliability is evaluated based on the overall success rate across five trials per item, as well as the consistency of processing times.

For the Level 1 benchmark, the experimental setup features simple objects with basic geometric shapes, including boxes, cylinders, and spheres. From left to right, the test objects comprise a water bottle, a sparkling beverage can, a cereal box, a toy egg-shaped object, a protein drink, and a Campbell’s soup can. These items are placed in upright positions and are within the operational width of the gripper. At this level, the system is required to detect each object, estimate an appropriate grasp point, and compute the corresponding robot configuration to execute the grasp with precision.

In greater detail, the robot first detects and classifies target items using a vision-based system. The initial estimation of the object’s location and pose is performed based on the image frame. The mobile platform then adjusts its position to achieve an optimal distance and orientation for the manipulator. Once the platform is correctly positioned, a second round of detection is conducted to obtain a more accurate estimation of the object’s position and rotation relative to the robot base.

Although this two-stage detection process introduces additional steps, it significantly enhances the precision and accuracy of object localization, which in turn contributes to a higher success rate in grasping tasks. After detection via the YOLOv11 model, the image is passed through a segmentation and image processing pipeline to extract object contours and estimate the object’s pose.

Subsequently, the object’s spatial information is transformed from the camera frame to the robot base frame to configure the robot’s end-effector pose. For the Level 1 benchmark, which only consists of upright items, the control primarily involves adjustments in the X-, Y-, and Z-axes, as well as the pitch angle.

To evaluate the system’s repeatability and reliability, the robot executes pick-and-place operations in a left-to-right order. Each item undergoes five independent trials, starting from different initial picking points. This procedure helps assess both the consistency of system performance and the robustness of the grasping pipeline across multiple repetitions.

According to the Level 1 benchmark results in Table 2, the Campbell item achieved the fastest execution time and the highest computation-to-success ratio (CSR), indicating superior computational and energy efficiency. In contrast, the protein drink exhibited the slowest execution time and the lowest CSR. Notably, the success rate for all items was 100%, demonstrating that the proposed method performs robustly on geometric object shapes in upright poses, enabling reliable 2D grasping.

Energy consumption, which is measured via the battery indicator, remained constant at 17 W, which is substantially lower than that of typical laptop or desktop power supplies exceeding 100 W. This highlights the efficiency of the proposed lightweight approach on embedded hardware. The average time for one frame and energy consumption are used to calculate the CSR. Importantly, the CSR serves as a comparative metric across platforms, balancing execution speed and energy use.

**Figure 18:**
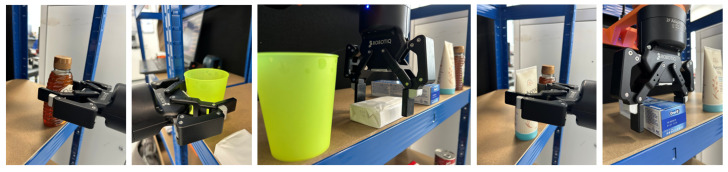
Grasping demonstration of Level 2 benchmark.

In the Level 2 benchmark, the object set includes more practical and irregularly shaped items: a plastic cup, a soap bar, a toothpaste box, a lotion bottle, and a honey bottle. These objects are presented in both upright and horizontal orientations to evaluate the system’s ability to adapt to varying object poses and orientations. Technically, the system must detect the object, estimate its pose, determine a viable grasp point, and compute the robot’s configuration, including both position and orientation, to successfully complete the grasping task as shown in Figure 18.

**Figure 19:**
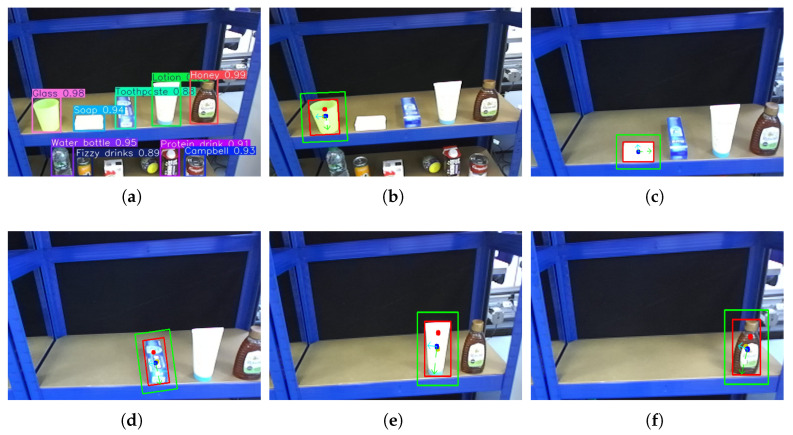
These are the detection and image processing results: (**a**) detection result from Yolov11n; (**b**) the object pose estimation of glass; (**c**) the object pose estimation of soap; (**d**) the object pose estimation of toothpaste; (**e**) the object pose estimation of lotion; (**f**) the object pose estimation of honey.

The process begins with detecting the target object in the image frame, identifying both its class and location. Subsequently, the system estimates the object’s pose as illustrated in Figure 19 and distance relative to the camera. Based on this information, the mobile platform navigates to a predefined approach position within the manipulator’s reachable workspace. Once the platform reaches this position, the system performs a secondary detection to verify the object’s location and refine the positional accuracy.

Using the updated object information, the system extracts the object’s X-, Y-, and Z-coordinates in the camera frame and transforms them into the robot’s base coordinate frame. The center point and an enhanced reference point on the object are then analyzed to determine whether the object is in an upright or horizontal (laying) orientation. This classification informs the configuration of the robot’s end-effector pose, including roll, pitch, and yaw. For example, if a soap bar is detected lying flat at a 90-degree orientation, the robot must approach the object with its pitch axis perpendicular to the surface and rotate its roll axis by 90 degrees. In contrast, for upright objects, the required motion primarily involves pitch adjustments and translation.

As shown in Table 3, the average processing time per item was under 5 s. The fastest case was 4.24 s (toothpaste), while the slowest was 5.55 s. The system achieved a 96% average success rate, with only one failure attributed to a missed detection of the toothpaste. This result suggests that incorporating more representative images of individual items into the training dataset could improve detection performance, as learning-based methods are highly dependent on the diversity and quality of the data.

**Figure 20:**
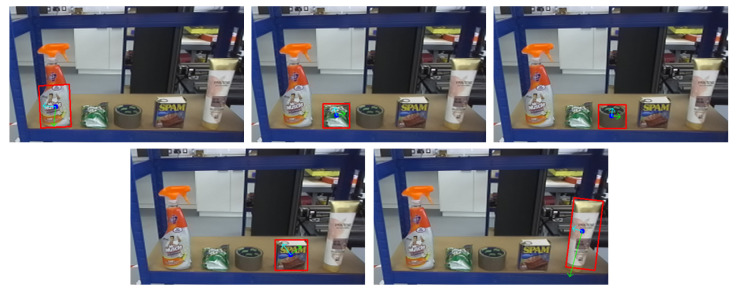
The results of level 3 benchmark and failures in terms of angle prediction from both arrows and grasping point by blue dot.

In the Level 3 benchmark, the proposed pose estimation method successfully grasped only the conditioner, which has a relatively simple shape but presents challenges due to its slight deformability, requiring careful control of gripper width and force. Other objects could not be grasped successfully, as the method lacks heuristic functions for identifying suitable grasping points. As illustrated in Figure 20, pixel-level centroid estimation alone is insufficient, often producing grasp points wider than the gripper width. Effective grasping of deformable objects such as the sweet bag and the conditioner further necessitates adaptive strategies, including force control and selection of top-surface or edge-based grasp points. These results indicate that while the proposed method is effective for rigid upright objects, further development is needed to incorporate grasp affordance reasoning for more complex and deformable items.

Compared to existing solutions [6], the proposed method requires only a single low-resolution RGB image and a limited number of point cloud samples as input. It demonstrates strong performance on geometric objects, particularly those in upright poses, where the problem is analogous to planar grasping. For smaller objects and those in laydown orientations with varied angles, successful grasps are still achievable; however, the accuracy of the Principal Component Analysis (PCA)-based angle estimation requires further improvement to enhance robustness across diverse object poses.

**Figure 21:**
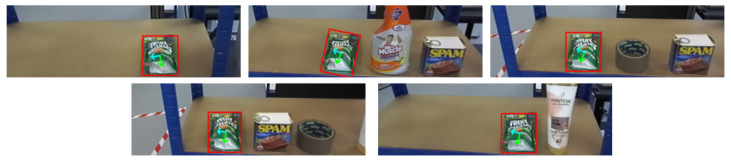
Empirical analysis on the proposed object pose estimation from the primary and secondary and the angle estimation.

The empirical robustness analysis of the proposed image-based grasping system is strongly influenced by the camera’s viewpoint. Ambiguity was observed for objects in a laydown pose, where the predicted angle deviated from the actual orientation, directly affecting roll control. Although the mobile platform assisted in aligning the camera with the target and an offset calibration was applied, further refinement is needed to achieve reliable angle estimation, as illustrated in Figure 21.

In contrast, upright objects achieved excellent results, requiring primarily pitch control, while yaw adjustment served only for obstacle avoidance and alternative approach paths. However, small objects, such as the egg-shaped toy, exhibited ambiguity due to their aspect ratio being similar to laydown objects, which led to incorrect pitch control. This suggests that dynamic aspect-ratio adaptation could improve performance.

In terms of physical properties, reflective and transparent objects remain unsuitable for image-based methods, as stereo and depth cameras often yield inaccurate point clouds. Regarding environmental factors, experiments were conducted under indoor artificial lighting, consistent with supermarket conditions. No significant lighting issues were observed, and robustness was supported by training with ±20% brightness variation.

Therefore, the main sources of uncertainty affecting robustness are laydown poses, small objects, and reflective or transparent surfaces. These remain challenges to be addressed in future work.

Overall, the experimental results demonstrate that the proposed system architecture is effectively optimized for both speed and task completion. Each component, namely object detection, pose estimation, transformation, and manipulation, contributes to a robust and efficient end-to-end pipeline suitable for real-world pick-and-pack applications.

## 4. Discussion

Overall, the pose estimation approach demonstrated satisfactory performance; however, it is not universally ideal and remains dependent on object characteristics. For instance, in the Level 1 benchmark, despite the overall task being successfully completed, the egg-shaped toy was incorrectly classified as being in a horizontal pose, although it was actually placed upright. This misclassification stems from the use of Principal Component Analysis (PCA), which estimates object orientation by calculating the dominant axis based on the distribution of pixels. Since the primary axis is derived from the object’s shape, smaller objects naturally result in shorter main axes.

In such cases, the enhanced point, which is computed along the primary axis, lies close to the centroid. Consequently, the spatial difference between the centroid and the enhanced point in the X, Y, and Z dimensions becomes minimal, often falling below the defined threshold used to distinguish between upright and laying poses. Specifically, for compact objects like the egg-shaped toy, extending the primary axis beyond the actual shape risks deviating from the physical object surface, potentially leading to inaccurate orientation estimation and unsuccessful grasps.

Nevertheless, this method is particularly effective for geometric and symmetric objects, offering accurate object pose estimation with low computational cost due to its straightforward processing pipeline. Its modular design enables easy extension to other applications, as it requires training only for the object detection module. The detection outputs, namely class labels and bounding box coordinates, are directly utilized in the segmentation and pose estimation stages without requiring prior object models or 3D data. This flexibility allows integration with a wide range of object detection algorithms and promotes adaptability to other domains, such as manufacturing and agriculture, where similar object characteristics are common.

The average execution time recorded in this study was approximately less than 5 s. While this may appear relatively long compared to other studies reporting grasp detection times in the range of a few milliseconds, it is important to consider several contextual factors. In this project, each stage of the process included image recording to document intermediate results and provide evidence, along with deliberate delay functions to ensure stability between stages. When these auxiliary functions were excluded, the core processing time was reduced to approximately 3 s.

A direct comparison with other works is inherently limited due to variations in experimental setups, including differences in CPU, GPU, RAM, communication protocols (e.g., wired vs. wireless), and the methods used to measure execution time. Unlike many prior studies that utilize high-performance computing platforms, this project emphasizes computational efficiency by deploying the system on an edge device. This design supports a wireless, compact, and energy-efficient architecture suitable for mobile manipulators powered by batteries.

Although wireless communication introduces additional latency, typically 2–3 times slower than wired connections, it offers greater flexibility and mobility. Each design choice carries inherent trade-offs. For ideal benchmarking, hardware configurations should be standardized to isolate and evaluate only the methodological differences. Nonetheless, the approach presented here offers a practical and energy-conscious foundation for the future development of lightweight robotic systems for real-world applications.

This benchmark focuses on image-based grasp detection and does not directly evaluate grasp stability or mechanical performance, which typically require separate analytic assessments. Qualitative and quantitative grasp analysis remains challenging; thus, task success is defined as the successful transfer of an item from the shelf to the destination, implicitly encompassing detection, planning, and execution. Although grasp stability is not explicitly measured, it is a critical factor influencing task reliability and must be considered in system design. Effective grasping requires both analytic and mechanical considerations to ensure stability. Ideally, the image-based centroid derived from visual perception should coincide with the actual physical centroid of the object, thereby minimizing torque and improving grasp stability. However, in this study such alignments could not be guaranteed due to camera viewpoint variations, image distortion, and the absence of physical object information available to the robot. Consequently, the proposed approach remains constrained to image-based centroid estimation, which may not always correspond to the true mass center of the object.

Hardware specifications significantly impact system performance and should be evaluated alongside methodological decisions. To address this, the compute-to-speed ratio (CSR) is used to capture both computational speed and energy efficiency. High processing speed alone is insufficient if it incurs excessive energy costs; real-world applicability demands efficient, resource-conscious computation.

While the current benchmark provides a foundation for evaluating vision-based grasping, it remains open to refinement and the inclusion of additional metrics. Broader adoption across heterogeneous platforms will enable more comprehensive evaluations of both physical and computational performance.

Future work could incorporate object-specific descriptors, grasp affordances, and depth imaging to enhance accuracy. Since all items are known in advance, object metadata could be stored and retrieved post-detection to reduce reliance on real-time estimation. Depth information would further improve pose estimation by validating surface consistency and enhancing grasp point selection beyond the limitations of RGB-based methods.

In addition, achieving higher accuracy in grasp point estimation may require 3D model fitting and point cloud analysis. Recent state-of-the-art approaches, such as random sample consensus (RANSAC), evolutionary algorithms, and the bees algorithm [34,35], provide improved real-time performance, consistency, generality, and robustness compared to purely image-based methods. While these approaches typically yield more reliable grasp points, their higher computational cost introduces a trade-off between accuracy and execution speed that must be carefully evaluated in real-world applications.

Finally, real-world deployment requires consideration of failure recovery and safety. The absence of tactile or in-hand sensors limits the robot’s awareness of object loss during transport. Multimodal sensing could improve perception. Moreover, integrating vision-based obstacle avoidance could proactively define restricted zones, supplementing built-in force protection for collaborative robots and supporting fully autonomous, remotely managed systems.

## 5. Conclusions

This study presents an end-to-end, computationally lightweight vision-based grasping system that demonstrates strong performance on a two-level pick-and-pack grocery benchmark. The system achieved a 100% success rate on Level 1 and 96% on Level 2, with an average execution time of under 5 s on an edge processing device, Jetson orin nano. Through careful optimization of inputs, parameters, and algorithmic methods, the system achieves real-time operation with low computational overhead and an average CSR of 0.0130, indicating energy efficiency.

The proposed framework consists of three key modules: object detection using YOLOv11n, instance segmentation via FASTSAM, and object pose estimation based on classical image processing techniques and Principal Component Analysis (PCA). The pose estimation approach is a novel contribution of this work, employing traditional mathematical methods without requiring prior grasping knowledge or data-intensive deep learning models. This results in a lightweight, interpretable, and efficient storage and energy solution that maintains high success rates while enabling rapid inference on compact, low-power edge devices.

Grasping points are determined based on the centroid derived from PCA, allowing the system to autonomously handle pick-and-pack tasks involving grocery items. The approach significantly reduces human intervention, labor costs, and order errors, offering a practical and scalable solution for smart cities. Furthermore, the modularity and generalizability of the system enable adaptation to other domains, such as logistics and manufacturing, depending on the properties of the target objects.

## Figures and Tables

**Table 1:** Denavit–Hartenberg Parameters.

Link *i*	$a_{i-1}$	$\alpha_{i-1}$	$d_{i}$	$\theta_{i}$
1	0	0	0	$\theta_{1}$
2	0	$\frac{\pi }{2}$	0.1807	$\theta_{2}$
3	−0.4784	0	0	$\theta_{3}$
4	−0.36	0	0	$\theta_{4}$
5	0	$\frac{\pi }{2}$	0.17415	$\theta_{5}$
6	0	−$\frac{\pi }{2}$	0.11985	$\theta_{6}$

**Table 2:** Results on the Level 1 benchmark.

Grocery Items	Weight (g)	Execution Times (s)	Average Time (s)	Success Rate	Energy Consumption (W)	CSR
Water bottle	526	4.40 4.19 4.64 4.51 4.41	4.430	100% (5/5)	17	0.0133
Sparkling can	268	4.73 4.33 4.47 4.47 4.43	4.486	100% (5/5)	17	0.0131
Cereal	32	4.48 4.62 4.47 4.39 4.44	4.480	100% (5/5)	17	0.0131
Egg-shaped toy	53	4.27 4.95 4.46 4.21 4.55	4.488	100% (5/5)	17	0.0131
Protein drink	387	5.65 4.63 4.24 4.88 4.36	4.752	100% (5/5)	17	0.0124
Campbell	337	4.49 4.30 4.33 4.31 4.34	4.354	100% (5/5)	17	0.0135

**Table 3:** Results on the Level 2 benchmark.

Grocery Items	Weight (g)	Execution Times (s)	Average Time (s)	Success Rate	Energy Consumption (W)	CSR
Glass	17	4.75 4.36 4.60 4.38 4.58	4.534	100% (5/5)	17	0.0130
Soap	95	5.14 4.96 4.38 4.37 4.43	4.656	100% (5/5)	17	0.0126
Toothpaste	130	4.24 4.45 5.55 - 4.78	4.755	80% (4/5)	17	0.0124
Lotion	167	4.38 4.47 4.58 5.00 4.56	4.598	100% (5/5)	17	0.0128
Honey	363	4.31 4.83 4.29 4.52 4.76	4.542	100% (5/5)	17	0.0130

## Data Availability

The original contributions presented in this study are included in the article; further inquiries can be directed to the corresponding author.
